# Models of Models: A Translational Route for Cancer Treatment and Drug Development

**DOI:** 10.3389/fonc.2017.00219

**Published:** 2017-09-19

**Authors:** Lesley A. Ogilvie, Aleksandra Kovachev, Christoph Wierling, Bodo M. H. Lange, Hans Lehrach

**Affiliations:** ^1^Alacris Theranostics GmbH, Berlin, Germany; ^2^Max Planck Institute for Molecular Genetics, Berlin, Germany

**Keywords:** preclinical models, computational model, mechanistic modeling, genetically engineered mouse models, transgenic mice, model optimization

## Abstract

Every patient and every disease is different. Each patient therefore requires a personalized treatment approach. For technical reasons, a personalized approach is feasible for treatment strategies such as surgery, but not for drug-based therapy or drug development. The development of individual mechanistic models of the disease process in every patient offers the possibility of attaining truly personalized drug-based therapy and prevention. The concept of virtual clinical trials and the integrated use of *in silico, in vitro*, and *in vivo* models in preclinical development could lead to significant gains in efficiency and order of magnitude increases in the cost effectiveness of drug development and approval. We have developed mechanistic computational models of large-scale cellular signal transduction networks for prediction of drug effects and functional responses, based on patient-specific multi-level omics profiles. However, a major barrier to the use of such models in a clinical and developmental context is the reliability of predictions. Here we detail how the approach of using “models of models” has the potential to impact cancer treatment and drug development. We describe the iterative refinement process that leverages the flexibility of experimental systems to generate highly dimensional data, which can be used to train and validate computational model parameters and improve model predictions. In this way, highly optimized computational models with robust predictive capacity can be generated. Such models open up a number of opportunities for cancer drug treatment and development, from enhancing the design of experimental studies, reducing costs, and improving animal welfare, to increasing the translational value of results generated.

## Mechanistic Models in Oncology

Despite major breakthroughs in cancer research and therapy, the disease still remains one of the world’s major healthcare challenges. A challenge that is exacerbated in Europe due to an aging population (1) and associated increase in cancer incidence (2). Ultimately, identification of successful therapies is hampered by the high level of complexity and genetic heterogeneity existing even within single tumor types, causing a large fraction of patients to remain refractory to treatment even with the most effective drugs we have available today (3–7).

Whenever we deal with complex problems mistakes are unavoidable, often with catastrophic consequences. Computer models can be used to simulate complex situations prior to testing in reality, allowing us to make these inevitable mistakes *in silico* and helping us to successfully avoid their deleterious impacts.

Although used in many areas from the aviation industry to climate prediction, a computational modeling strategy is still not being applied within two areas that have a fundamental impact on our health, wellbeing, and even our survival: drug-based treatment and drug development. Both are areas in which we still proceed statistically, prescribing drugs that ultimately only help a very small fraction of the patients receiving them and that could have negative consequences. Adverse drug effects lead to nearly 200,000 deaths per year in Europe (8), with enormous associated economic costs (9).

To overcome this problem, we have to be able to generate sufficiently accurate models of an individual patient—a virtual self—that allow us to observe the effects of different therapies. The basis is a generic computational model with the ability to predict effects and side effects of different drugs and their combinations, which is individualized based on a detailed characterization of a patient by molecular, sensor, and other techniques. In oncology, for example, we should ideally reflect the heterogeneity of the tumor by modeling individual tumor cells, including the stroma, to determine response. In addition, aspects of the liver should also be considered to determine the pharmacogenetics of the drug. Side effects can be evaluated by incorporating a selection of normal cell types and if we want to predict the effects and side effects of immunotherapies, we should also include elements of the immune system.

Currently, we are building large-scale mechanistic computational models of the signal transduction networks in cells (or cell collectives), based on the ever-expanding biological knowledge base, e.g., on signaling pathways in human cells. For this, we are using PyBioS, currently in its third iteration (PyBioS3), an integrated software platform for the design, modeling, and simulation of cellular systems (10, 11). The current model integrates about 50 cancer-related signaling pathways and makes use of a large and growing information base on functional consequences of genetic variants and mechanistic drug action. See Ref. (12–16), for further details of the modeling system and it applications.

To provide personalized predictions, the models are typically individualized with next generation sequencing-derived omics data (e.g., genome and transcriptome) from a patient and in the case of cancer also from individual tumors. For drug response predictions, the drugs to be “screened” are regarded as molecular entities that typically affect molecular networks. This information is translated into systems of ordinary differential equations, which can be solved numerically to make predictions regarding the functional response of the system in response to perturbations, such as specific genetic variants and/or drugs and their combinations. Adaptation of the model to biological observations and experimental data calls for optimization approaches. As the models become more complex, it is becoming increasingly important to use advanced parameter estimation strategies (17–20) to fit the model to the data. This can be done based on data generated on the types of preclinical models discussed in this issue.

Cancer is suited particularly well to this type of approach, as it is fundamentally a cellular disease. In tandem, high levels of funding for cancer research in the last decades has generated much of the knowledge required to establish generic computational models, such as information on the basic mechanisms of cancer and drug action, including molecular targets [e.g., (21–25)]. Diseased tissues can also be obtained as surgical or biopsy material. This means we can actually observe the changes—often dramatic—occurring in the tumor genome and transcriptome, making it easier to understand the likely functional consequences. Last but not least, computing power is now at a level (26) that makes predictive modeling on a large scale a realistic prospect.

## Predicting Uncertainty

The use of such computational models for personalizing medicine in the clinic does, however, still face a number of challenges. One of the main barriers to routine implementation of computational models in clinical scenarios is the accuracy of the prediction. Just how reliable can predictive computational models be?

The generic mechanistic model we have created integrates major molecular species, i.e., representations of genes, proteins, protein complexes, metabolites, etc., and biochemical/cellular processes, together making up an *in silico* representation of the cellular signaling network. Furthermore, it integrates modifications of the molecular species that are associated with cancer onset and development, such as mutated genes and proteins, reflecting gain-of-function or loss-of-function of oncogenes or tumor suppressor genes. Understanding how such a complex system functions as a whole is inferred from examination of its individual parts and their interactions [e.g., see Ref. (27, 28)].

To ensure that such mechanistic models are predictive we need a detailed assessment of the most important underlying biological reactions. However, within the large-scale networks generated, much of this information, such as binding affinities, (de)phosphorylation rates and synthesis, and degradation rates, is not easily obtained experimentally. To overcome the lack of information on parameters needed for this, we originally used a Monte Carlo strategy, selecting multiple random parameter vectors for multiple solutions (12). The unknown kinetic parameters are repeatedly sampled from probability distributions of values and used in multiple parallel simulations.

As the models grow in size, representing more signaling pathways and cellular components, so does the inherent complexity of the model and the associated number of unknown parameters involved in each process (e.g., kinetic constants, component concentrations). The signal transduction model we are currently working with comprises hundreds of genes, their modifications, and associated interactions, equating to tens of thousands of parameters. To infer the unknown parameters within this growing large-scale network, parameter optimization and reverse engineering strategies are used to increase the accuracy of predictions. This essentially means using data generated in experimental systems, e.g., mouse models, organotypic cultures, and cell culture, as well as data from patients (if available), for evaluating drug effects and other functional responses on the phenotypic and molecular level. We are generating this type of data within the scope of a number of national and international projects, such as the Horizon2020 project CanPathPro (www.canpathpro.eu); focused on the development of a combined experimental and systems biology platform for predictive modeling of cancer signaling, Treat20Plus, a German Federal Ministry of Education and Research funded project that uses a computational modeling approach to predict treatment outcome for metastatic skin cancer patients, and the recently concluded OncoTrack project [www.oncotrack.eu (15)], an IMI EU funded collaborative effort that aimed to develop and validate biomarkers for colon cancer, leveraging “virtual patient” models, and multi-level omics data to provide a personalized approach to the treatment of colon cancer. Using such data, we can train the model’s parameters and structure, and validate the predictions made in an iterative fashion.

When using models of such scale, we are faced with the problem that the number of unknown parameters significantly outnumbers the datasets that can be accumulated, leading to limited identifiability of parameters. To identify model parameters, statistical methods such as Bayesian and frequentist estimation as well as global and local optimization techniques can be applied (29, 30). Partitioning of the datasets into training, validation, and test sets, i.e., cross validation, facilitates identification of optimized parameter vectors and provides an unbiased estimate of how these vectors actually perform with an independent dataset (31). However, due to the limited number of training datasets available, overfitting is likely to occur, e.g., predictions become overly influenced by “noise” in the dataset rather than the inherent trends, resulting in poor predictive performance. A number of approaches can be used to overcome overfitting, such as increasing the amount of data (real or *in silico*) and/or using regularization parameters, but with complex models these strategies may bring limited improvements. An alternative strategy is to reduce the number of parameters by simplifying the model using model reduction methods (32, 33). These approaches face the challenge of identifying simplified models that exhibit dynamics comparable with the original. Ensembles of parameters that fit the data can further reduce overfitting effects. Moreover, techniques from artificial intelligence, such as deep learning, can help to learn directly from the data in an unsupervised fashion, without even knowing the underlying model (34).

## Models of Models: The Use of Preclinical Models for Optimizing *In Silico* Predictions

We see preclinical experimental models, including PDXs and transgenic mice [genetically engineered mouse models (GEMMs)] as well as cell and organotypic cultures, as being an integral part of the development and optimization of mechanistic computational models with more robust predictive capacity.

In particular, the contribution of mouse models to the understanding of fundamental biological processes, cancer research, and drug development has been significant, albeit with inevitable pitfalls (35, 36). PDX models have been shown to recapitulate the major molecular features of the tumor of origin, and therefore have immense utility in translational cancer research and personalized medicine applications (37, 38). Similarly, transgenic cancer mouse models, in which gene deletion or expression can be targeted in a spatial or temporal manner, are becoming an increasingly useful tool for understanding biological processes and disease development. These genetically engineered mice develop tumors *de novo*, which closely mimic both the histopathological and molecular features of human tumors, and provide an experimentally tractable *in vivo* platform for investigating disease mechanisms and determining response to therapies (36, 39).

While each preclinical system has its particular merits and pitfalls, it is clear they can provide a flexible and accurate experimental test bed for training and validating computational models. As part of a number of research projects (e.g., CanPathPro, Treat20Plus, and OncoTrack), the flexibility of preclinical systems is being leveraged to iteratively improve the accuracy of *in silico* predictions, regarding the functional consequences of molecular alterations on the signal transduction network, and the corresponding response to *in silico* drug treatment.

**Figure 1:**
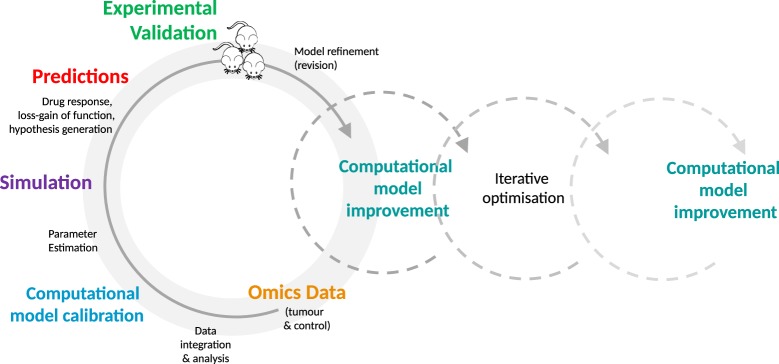
The iterative optimization cycle. From data integration and analysis to computational model development and optimization. Multi-tiered omics data generated from experimental models (e.g., mice or cell lines) are integrated into the generic signaling model and used to train the model. The estimated model parameters are then used to simulate the effect of perturbations, such as molecular alterations and drugs. The resulting predictions are validated in the experimental model by comparison with the expected values. This process is repeated on an iterative basis, enabling identification of key parameters, furthering mechanistic understanding of disease processes and drug action, and increasing the predictive accuracy of the model.

Even at the level of cell culture, an opportunity is provided to engage in a depth and breadth of experimentation that may not be feasible, cost and time-wise, using more complex preclinical models such as PDXs and GEMMs. Due to the simplicity and low cost of cellular systems, in-depth experimentation is made possible. A systematic comparison of predicted and observed responses of different cell models—in the case of oncology, tumor cells—to a variety of drugs and their combinations can be conducted in a quantitative and time-resolved manner. The observed phenotypic responses, e.g., does the cell respond to a drug or not, can be evaluated in the context of their specific molecular profiles. In addition, cell lines or organoids can be used for time-resolved analysis of the molecular changes (e.g., transcriptome, proteome, metabolome, and other omics-strategies) triggered by adding a drug or drugs under investigation. In-depth data are generated that is likely to be required for model optimization in high-dimensional parameter spaces. This detailed comparison of a cellular model at the phenotypic and molecular level with the predicted behavior of its computational avatar provides an essential data foundation that enables fine-scale optimization and increased predictive accuracy of *in silico* models. Computational models can be optimized in an iterative fashion through *in silico* perturbation experiments and subsequent validation of parameter information and functional response within experimental systems. See Figure 1, for a typical iterative workflow.

A stepwise process is taken in which the generic cellular signaling model is first adapted to the cancer being studied, with additional relevant pathways and mutations being added as modular elements [see (13, 16)]. Next, multi-tiered omics data from model samples (e.g., tumor and control samples from PDXs and transgenic mice or organotypic/tumor cell culture samples) is acquired and analyzed for alterations such as single nucleotide polymorphisms, gene fusions, and mutations. These data are integrated into the signaling network in modular format to individualize and calibrate the model.

Given that many parameters will be unknown, estimation strategies are applied [as detailed above and in Ref. (13, 16)] and the effects of perturbations (e.g., genetic variants and/or drugs), in combination with omics data (e.g., transcriptome of the respective samples), on network components are simulated. The generated simulation data are analyzed to make predictions regarding functional effects within the network, using indicators such as Myc levels or caspase activity as a proxy for phenotypic effects (e.g., cell proliferation, apoptosis). These predictions, including drug response, can subsequently be validated in an experimental system such as a PDX or cell culture. This detailed comparison of experimental results and predictions enables us to further refine our definition of the computational parameter space used for modeling, identifying parameters that are key to generating accurate predictions and that influence the phenotype of interest.

We are taking such an iterative approach within CanPathPro (www.canpathpro.eu), an EU Horizon2020 funded project, which focuses on the development of a combined experimental and systems biology platform for predictive modeling of cancer signaling. GEMMs, GEMM-derived cell lines, and organotypic cultures are used to provide an accurate route toward mapping the functional changes (e.g., deregulated pathways, pathway modules, and expression signatures) associated with a variety of mutated or over-expressed oncogenes and tumor suppressor genes that lead to different lung or breast cancer phenotypes.

For *in silico* experiments, a generic mouse-specific computational model has been generated based on the human-specific ModCell™ model (12, 13), leveraging the conservation and homologies existing between human and mouse genes, proteins, network structure, individual signaling relevant protein–protein interactions, and post-translational events (40–45). The mouse-specific computational model is then “personalized” with multi-layered omics data (e.g., exome, transcriptome, quantitative proteome, and phosphoproteome data), from individual tumor and control mouse tissues at different disease stages. This provides the model with essential information on parameters, such as presence or absence of mutations (including their frequencies), protein synthesis rates (derived from RNA-Seq data), and protein decay rates (e.g., derived from pulse chase experiments). Local and global optimization methods are employed to infer unknown parameters and ensembles of models with different parameter sets are used to simulate multiple hypothetical loss-/gain-of-function and under-/over-expression experiments or alternative interactions among the model components. These simulations lead to the formulation of testable hypotheses, such as determination of a specific cancer phenotype, pathway activity, and/or cross-talk. The perturbations that have the strongest effect on measurable read-out components are most likely to be used to reject or accept a hypothesis. Selected hypotheses can then be tested experimentally, first in cell lines and organotypic cultures and then in mouse models. Based on the outcome of these validation experiments, the quality and precision of the computational model predictions can be improved (see also Figure 1 for a depiction of the iterative process). These systematic and detailed investigations will enhance the design of experiments and facilitate identification of new mechanistic interactions as well as synergistic and cross-talk effects between the cancer pathways.

Overall, the abundance of data that can be generated within preclinical systems provides a platform for validating computational model predictions, identifying the areas of the parameter space that correspond most closely to reality. More accurate identification of this space can reduce the gap between prediction and reality, improving the ability of the model to make accurate predictions, and potentially improving the translational capacity of results for the human system.

## Models of Models: Accelerating Drug Development

The drug development pipeline is notoriously fraught with difficulties. Preclinical models are a pivotal part of this pipeline, bridging the translational gap from bench to bedside, however, approval rates of new drugs, especially in oncology, remain critically low—only ~5% of cancer drugs currently in Phase I trials will make it to market (46). A process with associated costs that amount to billions of dollars (47, 48). The reasons for such high attrition rates are complex and include the typically low response rate of patients to drugs, leading to the failure of late stage non-stratified clinical trials, usually after hundreds of millions to billions of dollars expenditure. Another key factor is the poor predictivity of preclinical models. Approximately 85% of drugs that have been successful in preclinical tests fail in early clinical trials, with cancer drugs making up the largest proportion of failures [reviewed in Ref. (49)]. Given that preclinical testing remains an integral part of the regulatory roadmap for drug development and approval, better approaches for improving the translational capacity of results generated are required.

In addition to virtual humans (either individual patients, or patients in large clinical trials), there is also the potential to model the large number of experimental models which are used during the preclinical phase of drug development. A highly optimized computational model opens up a range of opportunities for enhancing the design of experimental studies, thereby minimizing the number of experimental animals required, significantly reducing costs and improving animal welfare, and importantly, increasing the translational value of results generated.

The current strategy of directly extrapolating results of models to humans tends to ignore the enormous differences in the biology of models and human. It is really not very surprising that this will lead to imprecise predictions. After all, even the comparatively minor differences between different patients can cause enormous differences in response to already approved drugs (50). In a sense, we are trying to do the equivalent of directly transferring the results of a model plane in a wind tunnel to a large passenger plane, without taking into account key information on scaling effects and different aerodynamics.

To increase the translational success from experimental models to humans, we should first compare the results obtained from experimental models to the predictions obtained by computational modeling, e.g., the effects of a drug on a computer model of the animal or cell model, adapting this computer model first. This adaptation can then be transferred to the human model of each individual patient; an equivalent strategy to that used in airplane design.

**Figure 2:**
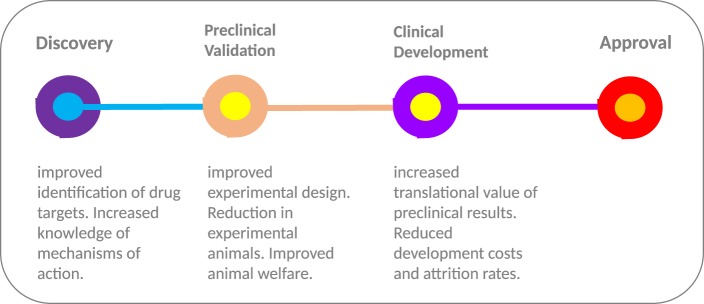
From discovery to approval. The use of computational models throughout the drug development process provides the scope to improve experimental design and increase the translational value of early and preclinical stage results. The “models of models” approach provides a flexible test bed, enabling extended testing not feasible in animal models due to welfare and economic concerns. In combination with highly optimized computational models with more robust predictive capacity, the approach has the potential to increase the translational value of preclinical results and improve the high level of drug attrition rates, especially within the cancer arena.

Deployment of *in silico* models at multiple stages throughout the drug development process (Figure 2) provides an opportunity to streamline the pipeline. During the early stages of drug development, *in silico* models can be deployed for selecting the most relevant drugs (and indeed models) for further development. Computational model-based knowledge gains in our understanding of the functional effects of disease-related molecular alterations could provide an effective pre-screening framework for selection of top priority candidates. By simulating the perturbation(s) occurring within the cellular transduction network, such as genetic alterations and drug treatment, *in silico* modeling has the capacity to improve understanding of disease progression and drug action. Model-based predictions of drug response/resistance and/or mode of action, based on the molecular profile of a specific experimental model, can in turn be independently validated in a preclinical experimental system using a variety of genetic manipulation techniques (e.g., RNA interference, over-expression analyses, etc.). In tandem, scope is provided to undertake experimental studies *in silico* that would not be possible due to cost and animal welfare constraints; for instance, more in-depth temporal investigations and extended screening of drug combinations.

A more robust translational route can be taken that combines the flexibility of optimized computational model predictions with experimental data, ultimately delivering benefits for patients.

## Outlook

Ultimately, the goal of any modeling approach is to generate results that will have a positive impact on patient outcomes and wellbeing, making it theoretically possible to identify the optimal therapy or preventive measure for every individual patient. Cancer models, whether experimental or computational, allow us to conduct investigations that are not possible on patients due to welfare, ethical, and economic considerations, generating information that will, in the short or long term, benefit patients. However, most model systems fail to fully recapitulate the human situation. In tandem with issues of experimental design, these inherent shortcomings will impact the translational value of models, exemplified by the high rates of attrition when preclinical findings are translated to clinical scenarios. It is also clear that existing computer models do not reflect the full complexity of the biological systems being simulated. Our own mechanistic models incorporate only a fraction of all human protein coding genes (ca. 2.5%) and do not reflect all aspects that play a role in a patient’s response to drugs, such as the immune system, metabolism, and the microbial milieu associated with tumors. As our knowledge accumulates on these aspects, including the molecular mechanisms underlying interactions between tumor cells, their surrounding soma cells, and infiltrating cells of the immune system, more complex computational models can be developed that reflect the true complexity and heterogeneity of tumors. We are continually working on these components, but for now take a pragmatic approach that has enabled the generation of a generic mechanistic model of a large-scale cellular signaling network capable of predicting patient-specific responses to miRNA-based treatments (14). Within the framework of a number of preclinical and clinical studies, issues of accuracy, sensitivity, and uncertainty are being addressed, as the model is expanded, optimized, and validated.

The tandem use of *in silico* and preclinical models—models of models—provides a necessary and complementary approach for improving the translational value of both model types. By leveraging the flexibility of experimental systems to generate datasets from multiple experimental set-ups, we have the potential to develop highly optimized and validated computational models, with robust predictive potential. This extends from improving the predictive accuracy of the *in silico* model through iterative rounds of experimentation and validation to enhancing preclinical experimental study design, opening up the possibility of minimizing the number of animals or experiments required, thereby improving animal welfare and reducing costs.

## Author Contributions

LO wrote the first draft of the manuscript. LO, AK, CW, BL, and HL contributed to the writing of the manuscript. LO, AK, CW, BL, and HL jointly developed the structure and arguments for the paper. All authors reviewed and approved the final manuscript.

## Conflict of Interest Statement

All authors are affiliated with Alacris Theranostics GmbH, which aims to develop “virtual patient” models for use in therapy choice and drug development. The reviewer, ML, and handling editor declared their shared affiliation.

## References

[1] European Commission. The 2015 Ageing Report. Economic and Budgetary Projections for the EU 28 Member States (2013–2060). Report No.: 3 Directorate-General for Economic and Financial Affairs, European Economy (2015).

[2] Ferlay J Soerjomataram I Ervik M Dikshit R Eser S Mathers C GLOBOCAN 2012 v1.0, Cancer Incidence and Mortality Worldwide: IARC CancerBase No. 11. Lyon, France: International Agency for Research on Cancer (2013).

[3] Gerlinger M Swanton C. How Darwinian models inform therapeutic failure initiated by clonal heterogeneity in cancer medicine. Br J Cancer (2010) 103(8):1139–43. 10.1038/sj.bjc.6605912 20877357 PMC2967073

[4] Meacham CE Morrison SJ. Tumour heterogeneity and cancer cell plasticity. Nature (2013) 501:328–37. 10.1038/nature12624 24048065 PMC4521623

[5] Burrell RA McGranahan N Bartek J Swanton C. The causes and consequences of genetic heterogeneity in cancer evolution. Nature (2013) 501:338–45. 10.1038/nature12625 24048066

[6] Fisher R Pusztai L Swanton C. Cancer heterogeneity: implications for targeted therapeutics. Br J Cancer (2013) 108(3):479–85. 10.1038/bjc.2012.581 23299535 PMC3593543

[7] Gerlinger M Rowan AJ Horswell A Math M Larkin J Endesfelder D Intratumor heterogeneity and branched evolution revealed by multiregion sequencing. N Engl J Med (2016) 366(10):883–92. 10.1056/NEJMoa1113205 PMC4878653 22397650

[8] European Commission. Proposal for a Regulation Amending, as Regards Pharmacovigilance of Medicinal Products for Human Use. Regulation (EC) No 726/2004. Impact Assessment. (2008). Available from: http://ec.europa.eu/health/files/pharmacos/pharmpack_12_2008/pharmacovigilance-ia-vol1_en.pdf

[9] Sultana J Cutroneo P Trifirò G Clinical and economic burden of adverse drug reactions. J Pharmacol Pharmacother (2013) 4:S73–7. 10.4103/0976-500X.120957 24347988 PMC3853675

[10] Wierling C Herwig R Lehrach H. Resources, standards and tools for systems biology. Brief Funct Genomic Proteomic (2007) 6(3):240–51. 10.1093/bfgp/elm027 17942476

[11] Klipp E Liebermeister W Wierling C Lehrach H Herwig R Systems Biology: A Textbook. Weinheim: Wiley-VCH GmbH & Co. KgaA (2009).

[12] Wierling C Kühn A Hache H Daskalaki A Maschke-Dutz E Peycheva S Prediction in the face of uncertainty: a Monte Carlo-based approach for systems biology of cancer treatment. Mutat Res (2012) 746(2):163–70. 10.1016/j.mrgentox.2012.01.005 22285941

[13] Wierling C Kessler T Ogilvie LA Lange BM Yaspo ML Lehrach H. Network and systems biology: essential steps in virtualising drug discovery and development. Drug Discov Today Technol (2015) 15:33–40. 10.1016/j.ddtec.2015.07.002 26464088

[14] Röhr C Kerick M Fischer A Kühn A Kashofer K Timmermann B High-throughput miRNA and mRNA sequencing of paired colorectal normal, tumor and metastasis tissues and bioinformatic modeling of miRNA-1 therapeutic applications. PLoS One (2013) 8(7):e67461. 10.1371/journal.pone.0067461 23874421 PMC3707605

[15] Henderson D Ogilvie LA Hoyle N Keilholz U Lange B Lehrach H. Personalized medicine approaches for colon cancer driven by genomics and systems biology: OncoTrack. Biotechnol J (2014) 9(9):1104–14. 10.1002/biot.201400109 25074435 PMC4314672

[16] Ogilvie LA Wierling C Kessler T Lehrach H Lange BM Predictive modeling of drug treatment in the area of personalized medicine. Cancer Inform (2015) 14:95–103. 10.4137/CIN.S1933 PMC4671548 26692759

[17] Banga JR Balsa-Canto E. Parameter estimation and optimal experimental design. Essays Biochem (2008) 45:195–210. 10.1042/BSE0450195 18793133

[18] Ashyraliyev M Fomekong-Nanfack Y Kaandorp JA Blom JG. Systems biology: parameter estimation for biochemical models. FEBS J (2009) 276(4):886–902. 10.1111/j.1742-4658.2008.06844.x 19215296

[19] Cedersund G Samuelsson O Ball G Tegnér J Gomez-Cabrero D Optimization in biology parameter estimation and the associated optimization problem. In: Geris L Gomez-Cabrero D, editors. Uncertainty in Biology: A Computational Modeling Approach. Cham: Springer (2016). p. 177–97.

[20] Penas DR González P Egea JA Doallo R Banga JR. Parameter estimation in large-scale systems biology models: a parallel and self-adaptive cooperative strategy. BMC Bioinformatics (2017) 18(1):52. 10.1186/s12859-016-1452-4 28109249 PMC5251293

[21] Hanahan D Weinberg RA The hallmarks of cancer. Cell (2000) 100(1):57–70. 10.1016/S0092-8674(00)81683-9 10647931

[22] Hanahan D Weinberg RA Hallmarks of cancer: the next generation. Cell (2011) 144(5):646–74. 10.1016/j.cell.2011.02.013 21376230

[23] Santos R Ursu O Gaulton A Bento AP Donadi RS Bologa CG A comprehensive map of molecular drug targets. Nat Rev Drug Discov (2017) 16(1):19–34. 10.1038/nrd.2016.230 27910877 PMC6314433

[24] Csermely P Korcsmáros T Kiss HJ London G Nussinov R. Structure and dynamics of molecular networks: a novel paradigm of drug discovery: a comprehensive review. Pharmacol Ther (2013) 138(3):333–408. 10.1016/j.pharmthera.2013.01.016 23384594 PMC3647006

[25] Karaman MW Herrgard S Treiber DK Gallant P Atteridge CE Campbell B A quantitative analysis of kinase inhibitor selectivity. Nat Biotechnol (2008) 26(1):127–32. 10.1038/nbt1358 18183025

[26] Processing Power Compared. Visualizing a 1 trillion-fold increase in computing performance. Experts Exchange. (2015). Available from: http://pages.experts-exchange.com/processing-power-compared/

[27] Kitano H Systems biology: a brief overview. Science (2002) 295(5560):1662–4. 10.1126/science.1069492 11872829

[28] Klipp E Liebermeister L Wierling C Kowald A Systems Biology. A Textbook. Weinheim: Wiley-Blackwell (2016). 504 p.

[29] Villaverde AF Banga JR Reverse engineering and identication in systems biology: strategies, perspectives and challenges. J R Soc Interface (2014) 11(91):20130505 10.1098/rsif.2013.0505 24307566 PMC3869153

[30] Fröhlich F Kaltenbacher B Theis FJ Hasenauer J Scalable parameter estimation for genome-scale biochemical reaction networks. PLoS Comput Biol (2017) 13(1):e1005331 10.1371/journal.pcbi.1005331 28114351 PMC5256869

[31] Hastie T Tibshirani R Friedman J The Elements of Statistical Learning: Data Mining, Inference and Prediction. 2nd ed New York: Springer Series in Statistics (2008). p. 241–9.

[32] Henriques D Villaverde AF Rocha M Saez-Rodriguez J Banga JR. Data-driven reverse engineering of signaling pathways using ensembles of dynamic models. PLoS Comput Biol (2017) 13(2):e1005379. 10.1371/journal.pcbi.1005379 28166222 PMC5319798

[33] Rao S Van der Schaft A Van Eunen K Bakker BM Jayawardhana B. A model reduction method for biochemical reaction networks. BMC Syst Biol (2014) 8(1):52. 10.1186/1752-0509-8-52 24885656 PMC4041147

[34] Angermueller C Pärnamaa T Parts L Stegle O. Deep learning for computational biology. Mol Syst Biol (2016) 12(7):878. 10.15252/msb.20156651 27474269 PMC4965871

[35] Day CP Merlino G Van Dyke T Preclinical mouse cancer models: a maze of opportunities and challenges. Cell (2000) 163(1):39–53. 10.1016/j.cell.2015.08.068 PMC4583714 26406370

[36] Kersten K de Visser KE van Miltenburg MH Jonkers J Genetically engineered mouse models in oncology research and cancer medicine. EMBO Mol Med (2017) 9(2):137–53. 10.15252/emmm.201606857 28028012 PMC5286388

[37] Schütte M Risch T Abdavi-Azar N Boehnke K Schumacher D Keil M Molecular dissection of colorectal cancer in pre-clinical models identifies biomarkers predicting sensitivity to EGFR inhibitors. Nat Commun (2017) 8:14262. 10.1038/ncomms14262 28186126 PMC5309787

[38] Morgan KM Riedlinger GM Rosenfeld J Ganesan S Pine SR Patient-derived xenograft models of non-small cell lung cancer and their potential utility in personalized medicine. Front Oncol (2017) 7:2 10.3389/fonc 28154808 PMC5243815

[39] van Miltenburg MH Jonkers J. Using genetically engineered mouse models to validate candidate cancer genes and test new therapeutic approaches. Curr Opin Genet Dev (2012) 22(1):21–7. 10.1016/j.gde.2012.01.004 22321988

[40] Kitano H. Biological robustness. Nat Rev Genet (2004) 5(11):826–37. 10.1038/nrg1471 15520792

[41] Pires-daSilva A Sommer RJ The evolution of signalling pathways in animal development. Nat Rev Genet (2003) 4(1):39–49. 10.1038/nrg977 12509752

[42] Cheng Y Ma Z Kim BH Wu W Cayting P Boyle AP Principles of regulatory information conservation between mouse and human. Nature (2014) 515(7527):371–5. 10.1038/nature13985 25409826 PMC4343047

[43] Stergachis AB Neph S Sandstrom R Haugen E Reynolds AP Zhang M Conservation of trans-acting circuitry during mammalian regulatory evolution. Nature (2014) 515(7527):365–70. 10.1038/nature13972 25409825 PMC4405208

[44] Tan CS Bodenmiller B Pasculescu A Jovanovic M Hengartner MO Jørgensen C Comparative analysis reveals conserved protein phosphorylation networks implicated in multiple diseases. Sci Signal (2009) 2(81):ra39. 10.1126/scisignal.2000316 19638616

[45] Kiel C Aydin D Serrano L. Association rate constants of ras-effector interactions are evolutionarily conserved. PLoS Comput Biol (2008) 4(12):e1000245. 10.1371/journal.pcbi.1000245 19096503 PMC2588540

[46] Thomas DW Burns J Audette J Carroll A Dow-Hygelund C Hay M Clinical Development Success Rates 2006–2015. Biotechnology Innovation Organization (BIO) (2016). Available from: https://www.bio.org/sites/default/files/Clinical Development Success Rates 2006-2015 - BIO, Biomedtracker, Amplion 2016.pdf [Accessed 30 May 2017].

[47] Herper M The Cost of Creating a New Drug Now $5 Billion, Pushing Big Pharma to Change. New Jersey: Forbes, Pharma & Healthcare (2013). Available from: https://www.forbes.com/sites/matthewherper/2013/08/11/how-the-staggering-cost-of-inventing-new-drugs-is-shaping-the-future-of-medicine/#3df7a07e13c3

[48] DiMasi JA Grabowski HG Hansen RW Innovation in the Pharmaceutical Industry: New Estimates of R&D Costs. Boston: Tufts Center for the Study of Drug Development (2014). Available from: http://csdd.tufts.edu/news/complete_story/cost_study_press_event_webcast

[49] Mak IW Evaniew N Ghert M. Lost in translation: animal models and clinical trials in cancer treatment. Am J Transl Res (2014) 6(2):114–8. 24489990 PMC3902221

[50] Schork NJ Personalized medicine: time for one-person trials. Nature (2015) 520(7549):609–11. 10.1038/520609a 25925459

